# Validation of 11 added items of the outpatient version of the Utrecht Symptom Diary in patients receiving chemotherapy or targeted therapy

**DOI:** 10.1186/s41687-024-00794-w

**Published:** 2024-10-18

**Authors:** Josephine J. Koldenhof, Bernice O. Akpobome, Danielle Zweers, Stance Klaasse, Saskia C. C. M. Teunissen, Petronella O. Witteveen, Karijn P. M. Suijkerbuijk, Alexander de Graeff, Frederieke H. van der Baan

**Affiliations:** 1 Department of Medical Oncology, University Medical Centre Utrecht, University Utrecht, (room B02.225), PO Box 85500, Utrecht, 3584 CX The Netherlands; 2 Center of Expertise in Palliative Care, Julius Center for Health Sciences and Primary Care, University Medical Centre Utrecht, University Utrecht, Utrecht, The Netherlands; 3 Department of Haematology, University Medical Centre Utrecht, University Utrecht, Utrecht, The Netherlands

**Keywords:** Cancer, Chemotherapy, Patient-reported outcome measurement, Supportive care, Symptom assessment, Targeted therapy

## Abstract

**Introduction:**

The Utrecht Symptom Diary (USD) is a validated Dutch patient-reported outcome measurement (PROM) tool - based on the Edmonton Symptom Assessment System - to assess and monitor symptoms in cancer patients. The USD contains 11 items concerning frequently occurring symptoms in cancer patients (pain, sleeping problems, dry mouth, dysphagia, lack of appetite, abnormal stool, nausea, shortness of breath, fatigue, anxiety and depressed mood) and an item on overall well-being. For the outpatient USD 11 items concerning frequently occurring signs and symptoms in patients receiving chemotherapy and/or targeted therapy were added to the USD: taste alteration, oral pain, weight loss, diarrhoea, hair changes, skin problems, nail problems, eye problems, tingling, concentration problems and problems with sexuality. This current study aimed to evaluate the 11 added items on this treatment specific outpatient USD in cancer patients receiving intravenous chemotherapy and/or targeted therapy.

**Methods:**

Observational longitudinal retrospective cohort study including all adult outpatients with cancer receiving intravenous chemotherapy and/or targeted therapy in an academic hospital in the Netherlands who completed at least one outpatient USD as part of routine care (2012–2021). Relevance, comprehensiveness as well as criterion and construct validity were assessed.

**Results:**

1733 patients who completed ≥ 1 outpatient USD during intravenous chemotherapy and/or targeted therapy were included for analysis. Relevance as well as comprehensiveness of the items on the outpatient USD in this patient population was shown. Criterion validation was demonstrated for all added items of the outpatient USD – except for the item on oral pain. An additional analysis showed that mouth problems were detected with both outpatient USD items oral pain and dry mouth. Construct validity was demonstrated for the items hair changes and skin and nail problems. Construct validity on eye problems was not tested due to the low number of paired outpatient USDs.

**Conclusions:**

The treatment specific outpatient USD is a validated PROM in outpatients with cancer receiving intravenous chemotherapy and/or targeted therapy. Considering its validity in this broad group of patients, we think the treatment-specific outpatient USD is widely applicable. In addition to providing tailored supportive symptom care, the USD-data can be used to increase knowledge about symptom burden in daily practice in this population.

**Supplementary Information:**

The online version contains supplementary material available at 10.1186/s41687-024-00794-w.

## Introduction

During treatment with chemotherapy and/or targeted therapy cancer patients experience multiple symptoms associated with disease and/or treatment [1, 2]. Poorly controlled symptoms can lead to decreased health related quality of life (HRQL), treatment modifications, visits to the emergency department and hospitalization [3]. The use of patient reported outcome measurement (PROMs) tools facilitates identification and management of symptoms [4] and patients’ needs [5]. The Edmonton Symptom Assessment System (ESAS) is a validated and frequently used patient-reported outcome measurement tool (PROM) to assess and monitor symptoms in both clinical practice and research [6, 7].

The Utrecht Symptom Diary (USD) is an adapted Dutch translation of the ESAS including 12 items: pain, sleeping problems, dry mouth, dysphagia, lack of appetite, abnormal stool, nausea, shortness of breath, fatigue, anxiety, depressed mood and overall well-being. The USD is used in daily practice in several hospitals, general practices, and hospices in the Netherlands and use of the USD is recommended by the Netherlands Quality Framework for Palliative Care [8]. A recent study has demonstrated the validity of the USD in cancer patients in both the curative and palliative phase of disease and capable of detecting clinically important changes over time [9].

A brief, relevant and comprehensive PROM is essential, since patients may become demotivated when PROMs are too long or when symptoms patients find relevant are missing [3, 10]. Therefore, multiple modules of the USD have been developed, targeting specific tumours [11, 12] or treatments [13]. Based on the literature and clinical experience the outpatient USD was developed for patients receiving intravenous chemotherapy and/or targeted therapy (Appendix A). For the treatment-specific outpatient USD 11 items concerning frequently occurring signs and symptoms in patients receiving chemotherapy and/or targeted therapy were added to the USD: taste alteration, oral pain, weight loss, diarrhoea, hair changes, skin problems, nail problems, eye problems, tingling, concentration problems and problems with sexuality [1, 14, 15]. The use of the outpatient USD has been part of routine care for more than a decade. Since 2015 the USD has been integrated into the electronic medical files (EMF).

This study aimed to evaluate the validity of the added items on the treatment-specific outpatient USD in patients receiving intravenous (IV) chemotherapy and/or targeted therapy in the outpatient clinic. In the following, for the sake of brevity the term ‘outpatient USD’ will be used.

## Methods

### Patient population and setting

In this observational longitudinal cohort study, all adult (≥ 18 years) outpatients with cancer treated with IV chemotherapy and/or targeted therapy and who completed at least one outpatient USD during treatment between June 2012 and January 2021 were included for retrospective analysis. Patients were treated with curative or palliative intent at the Department of Medical Oncology, University Medical Center (UMC) Utrecht, the Netherlands. Patients were routinely offered an outpatient USD to assess and monitor symptoms and their burden. They completed the outpatient USD on paper or in the patient portal of the EMF. To tailor care, oncology nurses discussed the clinically relevant USD scores (≥ 3) with the patient.

Patients were offered an opt out if they did not want their USD data to be used for research purposes. This research was not considered subject to the Medical Research Involving Human Subjects Act by the institutional review board of the UMC Utrecht (METC number 15/087).

### Data collection

The outpatient USD consists of 23 items and does not assess an underlying construct (Appendix A). The items were scored using an 11-point numerical scale (a higher score indicating a higher intensity) [9]. Patients were asked to score each item considering symptoms experienced since the last visit to the outpatient clinic. Alongside the 23 items, patients could add symptoms as an open text and were invited to assign priority to symptoms requiring supportive care. This invitation enhanced patients’ autonomy and facilitated their ability to make their own choices and take control of their health care during discussions with their healthcare professional. All outpatient USDs completed during treatment up to three weeks after the last treatment were included, as this is the time frame in which treatment-related symptoms are expected to occur. In this study we focused on the previously mentioned added items on symptoms on the outpatient USD. Pseudonymized data on patient characteristics, disease, treatment, and USD scores were collected retrospectively from the EMF.

We also used data from the distress thermometer and problem checklist (DT&PC) which was routinely offered to patients at the start of every treatment and when clinically indicated. The DT&PC is a screening tool which is recommended to detect psychosocial care needs [16, 17]. The validated distress thermometer (DT) is a self-report measure of distress (Visual Analogue Scale 0–10; a higher score indicating more distress). The problem checklist (PC) identifies problems that cause distress across five domains: physical, practical, family, spiritual, and emotional. Patients are asked to indicate (Yes/No) whether the item has been a problem in the past week including the day it was completed [16, 18]. Patients reporting a higher number of problems score higher on the DT [19]. Data on the DT&PC were also used from the EMF.

Descriptive statistics were obtained to summarize the characteristics and symptoms of the patients. R software for statistical computing was used for statistical analysis (R version 4.0.3 Package epitools). Patients with a missing value for a studied USD item were excluded for the analysis of that item. The percentage of missing items is presented for each item.

Definitions, methods, and quality criteria for the measurement properties of the outpatient USD were based on the COnsensus-based Standards for the selection of health Measurement INstruments (COSMIN) initiative [20, 21].

## Validation

### Relevance and comprehensiveness

To study the relevance of all items in the outpatient USD, we selected the highest score for each patient given to each item during treatment. We considered an item to be relevant when a symptom was scored prevalent (USD score ≥ 1) by at least 10% of the patients at least once during the treatment trajectory [22].

To investigate the comprehensiveness of the outpatient USD, we analyzed the symptoms and problems that were added as open text by the patients during treatment. The open responses were reviewed, categorized in overarching symptoms and discussed to reach agreement by clinicians (AG, DZ, JK, SK) who are familiar with the USD. First one or more symptoms/problems were extracted from an open response e.g. *‘dizzy especially when standing up and pounding heart’* into dizziness and palpitations. Thereafter symptoms/problems were grouped e.g. nasal dryness, nasal pain and nasal congestion were grouped into nasal problems (Appendix C). Inclusion of a symptom in the outpatient USD was considered when added by ≥ 10% of patients.

### Criterion validity

Criterion validity is the degree to which the scores of the outpatient USD are an adequate reflection of the gold standard [21]. In the absence of a true gold standard, the DT&PC was used as a reference standard. Criterion validation was analyzed only for the outpatient USD items that were not previously validated [9] and for which a corresponding item was present on the DT&PC. These included: taste alteration, weight loss, diarrhea, oral pain, tingling, concentration problems and problems with t sexuality.

Per patient we selected the first DT&PC and outpatient USD completed ≤ 1 day, during treatment. In line with the previous USD validation study we used the cutoff point ≥ 3 as a clinically relevant cutoff score [9, 23–25], resulting in a dichotomous variable which was compared to the DT&PC dichotomized responses (problem Yes/No). For each item an area under the curve (AUC) of the receiving operating curve (ROC) was calculated and an AUC ≥ 0.70 was interpreted as an adequate reflection of the DT&PC [26]. We next performed a subsequent analysis combining the outpatient USD items oral pain and dry mouth, in which we considered the USD positive if at least one of these items scored ≥ 3. This combined variable (1/0) was compared to mouth sores on the DT&PC. Stratified bootstrap replicates of 2000 were used to compute the corresponding 95% confidence interval of the AUC [27].

### Construct validity

Construct validity is the degree to which outpatient USD scores are consistent with hypotheses (for instance comparisons with the situation before start of treatment ) formulated before data analysis [21]. Hypotheses were formulated based on literature and expert opinions of the clinicians for four items on the outpatient USD with no corresponding item on the DT&PC which were: hair changes, and nail, skin and eye problems (Table 1).

Using the McNemars chi-squared test, a paired comparison was done on the outpatient USD completed closest before start treatment against those completed at a the first USD available during treatment, in the time frame the symptom was expected to occur. This time frame was based on the literature and clinical experience and specified in the hypothesis. A hypothesis was only tested if at least 100 paired USDs were available [28]. A level of significance of 5% was used to confirm or reject the test. Again, we used the cutoff ≥ 3 to define a clinically relevant USD score.

**Table 1:** Hypotheses

Outpatient USD item	Hypotheses
Hair changes	“Patients using anthracyclines, taxanes (paclitaxel, nab-paclitaxel, docetaxel) and irinotecan for at least six weeks experience hair changes more often than before treatment” [29, 30]
Nail problems	“Patients using panitumumab, cetuximab, docetaxel, paclitaxel, and/or nab-paclitaxel for at least eight weeks experience nail problems more often than before treatment” [14, 31, 32]
Skin problems	“Patients using capecitabine for at least six weeks experience skin problems more often than before treatment” [31, 33, 34].
Eye problems	“Patients using trastuzumab, cetuximab and/or panitumumab for at least seven weeks experience eye problems more often than before treatment” [15, 35]

## Results

Between June 2012 and January 2021 2164 patients completed at least once an outpatient USD either before or during treatment at the outpatient clinic.

Table 2 shows the characteristics of the 1733 patients who completed at least one outpatient USD during the selected treatment of IV chemotherapy and/or targeted therapy (up to 3 weeks after the last dose) and were included for analysis: 1147 (66%) received chemotherapy without targeted therapy, 586 (34%) received at least once targeted therapy with or without chemotherapy. Appendix B gives an oversight of the different treatment regimens. Of these patients, 17% completed one outpatient USD, 54% completed 2–5 outpatient USDs and 28% ≥6 outpatient USDs during this treatment.

In order to estimate the representativity of our study population for all outpatients receiving IV chemotherapy and/or targeted therapy, we analyzed what percentage of patients completed at least one USD. We found that in 2019 95% (575/608) patients treated with chemotherapy and/or targeted therapy completed the outpatient USD at least once.

**Table 2:** Patient characteristics

	*N* = 1733	
Age, median [IQR]		61 [52–70]
Gender, N (%)	Female	950 (55)
Treatment, N (%)	Chemotherapy	1147 (66)
	Targeted therapy	225 (13)
	Chemotherapy and targeted therapy	361 (21)
Primary cancer site, N (%)	Digestive tract	829 (48)
	Female genital tract	365 (21)
	Breast	210 (12)
	Head and neck	120 (7)
	Male genital tract	86 (5)
	Central Nervous System	58 (3)
	Other	65 (4)

### Relevance

All items of the outpatient USD that had not been validated previously - taste alteration, oral pain, weight loss, diarrhoea, hair changes, skin problems, nail problems, eye problems, tingling, concentration problems and problems with sexuality - were prevalent (USD score ≥ 1) in ≥ 10% of the population with prevalence ranging from 42% for nail problems to 76% for taste alteration. Additionally, all items were scored as clinically relevant (score ≥ 3) at least once during treatment by ≥ 10% of the patients with percentages ranging from 22% for nail problems to 58% for taste alteration. For all items, the percentage of missings was low; the highest percent missing was found for problems with sexuality which was 3% (Table 3).

**Table 3:** Relevance

Items, *N* (%)	*N* = 1733		
	Prevalent	Clinically relevant	Missing
	USD score ≥ 1	USD score ≥ 3	
Taste alteration	1324 (76)	1008 (58)	10 (0.6)
Oral pain	897 (52)	514 (30)	3 (0.2)
Weight loss	1227 (71)	842 (49)	7 (0.4)
Diarrhea	1134 (65)	805 (46)	4 (0.2)
Hair changes	1094 (63)	803 (46)	8 (0.5)
Skin problems	1053 (61)	682 (39)	6 (0.3)
Nail problems	729 (42)	376 (22)	4 (0.2)
Eye problems	865 (50)	497 (29)	5 (0.3)
Tingling	1037 (60)	722 (42)	5 (0.3)
Concentration problems	1159 (67)	787 (45)	3 (0.2)
Problems with sexuality	739 (43)	489 (28)	58 (3)
USD = Utrecht Symptom Diary			

### Comprehensiveness

Of the 1733 unique patients, 824 patients added 1819 unique symptoms/problems as an open text with an average of 1 per patient. Unique symptoms/problems were categorized into 177 symptoms/problems. The symptoms pain and fatigue were added even though they were already an item on the outpatient USD by 23.3% and 6.9% of the patients, respectively. Table 4 shows the symptoms (including specific pain subcategories) identified from the open responses with a prevalence of at least 2%. Since they occurred in < 10% of the patients, we decided not to add them to the outpatient USD.

**Table 4:** Comprehensiveness, extra symptoms added by patients to the outpatient USD

Symptoms	*N* (%)
Cough	37 (2.0)
Dizziness	59 (3.2)
Itching	39 (2.1)
Pain, subcategories	
– Headache	54 (3.0)
– Muscle pain	47 (2.6)
– Back pain	46 (2.5)
– Abdominal pain	36 (2.0)

## Criterion validity

During treatment until three weeks after treatment completion, 971 patients completed a USD and DT&PC with a ≤ 1 day interval.

Table 5 shows the results on criterion validity. Dichotomous USD scores based on the previously defined cutoff of 3 were compared to the presence of a problem on the DT&PC. Except for oral pain - with an AUC of 0.63 - all the items showed a good criterion validity with an AUC of ≥ 0.70. Reconsidering, we noticed patients who reported mouth problems as a problem on the DT&PC and did not experience oral pain were likely to have experienced dry mouth. The subsequent analysis comparing the USD items dry mouth and/or oral pain with mouth problems on the DT&PC showed a sensitivity of 0.75 and specificity of 0.80. For all items there were < 10% missing values.

**Table 5:** Criterion validity

Symptoms on USD	*N* = 971							
	DT&PC *N* (%)		Outpatient USD *N* (%)		Sensitivity	Specificity	AUC [95% CI]	
	Yes	Missing	≥ 3	Missing				
Taste alteration	146 (15)	10 (1)	181 (19)	22 (2)	0.71	0.90	0.81 [0.77–0.85]	
Weight loss	272 (28)	10 (1)	249 (26)	16 (2)	0.70	0.92	0.81 [0.78–0.84]	
Diarrhoea	150 (15)	12 (1)	148 (15)	12 (1)	0.75	0.96	0.85 [0.82–0.89]	
Oral pain	83 (9)	14 (1)	56 (6)	11 (1)	0.29	0.97	0.63 [0.58–0.68]	
Tingling	132 (14)	10 (1)	116 (12)	10 (1)	0.67	0.97	0.82 [0.78–0.86]	
Concentration problems	221 (23)	11 (1)	208 (21)	10 (1)	0.61	0.90	0.76 [0.72–0.79]	
Problems with sexuality	79 (8)	20 (2)	147 (15)	68 (7)	0.81	0.89	0.85 [0.80–0.89]	

*USD* utrecht symptom diary; *DT&PC* distress thermometer and problem checklist; *AUC* area under the curve; *CI* confidence interval

## Construct validity

The number of patients with USDs corresponding to the hypothesis on the hair changes and the nail, skin and eye problems were 392, 273, 170 and 76 respectively for each of the comparison groups.

Table 6 shows how the data correlate with the hypothesis generated. Up to 11% of the patients scored an item as present at baseline already. Construct validity was demonstrated for the items on hair changes, and nail and skin problems. For the hypothesis on eye problems we had insufficient (< 100) paired outpatient USDs to test the construct validity.

**Table 6:** Construct validation

Symptoms		USD score ≥ 3 *N* (%)	*P*-value^a^
Hair changes (*N* = 392)	Before treatment	45 (11)	< 0.0001
	During anthracyclines, paclitaxel, docetaxel or irinotecan treatment	204 (52)	
Nail problems (*N* = 273)	Before treatment	21 (8)	0.004
	During cetuximab, panitumumab, docetaxel or paclitaxel treatment	41 (15)	
Skin problems (*N* = 170)	Before treatment	19 (11)	0.0004
	During capecitabine	43 (25)	

*USD* utrecht symptom diary

^a^McNemars chi-squared test

## Discussion

We developed the outpatient USD in which items were added to the core version of the USD in order to early recognize and monitor symptoms in outpatients receiving intravenous chemotherapy or targeted therapy. With this large cohort study we assessed the relevance and comprehensiveness of the items as well as the criterion and construct validity of 11 additional items. Construct validity of the item on eye problems could not be tested due to a low number of paired USDs.

However, since the USD is part of routine care for over a decade and contains a question on missing symptoms, the routinely collected data offers the opportunity to test comprehensiveness as well as relevance of the instrument.

## Relevance

Relevance of the items was demonstrated since all additional items on the outpatient USD were prevalent (USD score ≥ 1) and clinically relevant (USD score *≥* 3) in ≥ 10% of the population. These items are in line with the findings by others who also identified these items as frequently occurring and/or being burdensome symptoms in patients treated with chemotherapy and/or targeted therapy [1, 2]. In their literature synthesis, Reilly et al. [1] found that weight loss, concentration problems and numbness/tingling were reported by ≥ 40% of the patients, sexual dysfunction and taste alteration by 32%, hair loss/appearance by 21%, diarrhoea by ≈ 15% and skin changes by ≈ 13%. Nail and eye problems are frequently reported by patients and healthcare professionals in other studies [15, 32].

## Comprehensiveness

The outpatient USD was found to be complete since none of the additional symptoms/problems in the open responses was reported by ≥ 10% of the patients.

Reilly et al. [1] reported a high prevalence of nocturia (75%) and cough (52%) in 2/21 studies included in their pooled analysis. In these two studies most included patients were diagnosed with lung cancer. We considered these symptoms to be irrelevant to the outpatient USD for our study population since they were added by none (nocturia) or few (cough) of the patients in our population, which did not include lung cancer patients.

## Criterion validation

We found a good criterion validation for the items taste alteration, weight loss, diarrhoea, tingling, concentration problems and problems with sexuality, using the DT&PC as reference standard. The criterion validation on the item oral pain – mouth problems on the DT&PC - could initially not be demonstrated. In a subsequent analysis, validating oral pain and dry mouth together on the DT&PC mouth problems item, we found an improved sensitivity and specificity. This implies that patients who report a mouth problem on the DT&PC mainly experience oral pain or dry mouth (or a combination of both) which is thus detected by the outpatient USD.

## Construct validation

The items on nail and skin problems and on hair changes had good construct validity. Skin problems occur in 50–100% of the patients, hair changes in up to 50% of the patients and nail problems in 10–20% of the patients undergoing intravenous chemotherapy and/or targeted therapy [14, 29, 31, 33, 36].

Ali et al. [37] described ocular toxicities as commonly occurring in patients receiving cetuximab such as dry eyes (67%), blepharitis (63%), conjunctivitis (10–18%) and eyelid rash (38%) in patients receiving cetuximab and watery eyes (21%) in patients receiving trastuzumab. In our study population we had a low number of paired USDs of patients receiving trastuzumab, cetuximab and/or panitumumab scoring the item eye problems ≥ 3. As a result, we could not assess the hypothesis for the construct validity on the item eye problems. Further research on this item using a higher sample size is needed to test its construct validity [21].

## Strengths and limitations

A strength of this study is its representation of the population of outpatients with different cancer diagnoses, since all patients receiving intravenous chemotherapy and/or targeted therapy who at least completed one USD were included. We showed that the use of the outpatient USD is feasible since 95% of the patients completed at least one USD during treatment. Moreover we found a low number of missing items. Furthermore, comparison was made between the outpatient USD and the DT&PC completed within ≤ 1 day which enabled the comparison of symptoms that arose within the same period thus reducing the risk of bias. This was also reported by COSMIN as essential for good criterion validation [21]. In line with our previous validation study [9] we consider the comparison of the outpatient USD to a concurrent DT&PC as a strength since patients scored a symptom as present or not on the DT&PC.

A limitation is that we have only data on USDs and DT&PCs when offered and completed since we used routine clinical data which were retrospectively analyzed. As a result we were not able to examine test-retest reliability and to assess content validity. Patients completed the outpatient USD when visiting the outpatient clinic which often is ≥ 21 days apart. To examine test-retest reliability two USDs completed within a few days from each other should have been available to rule out any event that would have influenced the patient’s USD score, for example toxicity from treatment. Furthermore, since the USD is part of routine care for over a decade and contains a question on missing symptoms, the routinely collected data did offer the opportunity to test relevance and comprehensiveness of the items. However, to assess content validity during PROM development it is recommended to conduct interviews and/or focus groups [21]. We therefore will perform a separate prospective study on reliability and content validity in the future.

Secondly, despite the fact that we think that our study population is a true representation of the percentage of patients receiving intravenous chemotherapy and patients receiving intravenous targeted therapy it should be noted that the majority of patients in our study were treated with chemotherapy and to a lesser extent with targeted therapy.

Lastly, we found that some of the patients scored an item as present at baseline already, most probably due to disease related symptoms, persistent symptoms due to prior treatments, or symptoms related to comorbidity such as an ocular or skin disease. This may have influenced our results.

## Conclusion

Our analyses support the validity of the outpatient USD in measuring the most prevalent symptoms that affect outpatients with cancer treated with intravenous chemotherapy and/or targeted therapy. Although further research may clarify our findings on the item eye problems we think that the outpatient USD is widely applicable since it is validated in a broad group of patients with cancer in terms of cancer diagnosis and anticancer treatments. Furthermore, USD data can be used to increase knowledge about symptom burden to optimize supportive symptom care in daily oncology practice in this population.

## Electronic supplementary material

Below is the link to the electronic supplementary material.

Supplementary Material 1

Supplementary Material 2

Supplementary Material 3

## Data Availability

The datasets used and/or analysed during the current study are available from the corresponding author on reasonable request.

## Abbreviations

AUC: area under the curve
COSMIN: COnsensus-based Standards for the selection of health Measurement INstruments
DT&PC: distress thermometer and problem checklist
IV: intravenous
PROM: patient-reported outcome measurement
ROC: receiving operating curve
USD: Utrecht Symptom Diary

## References

[1] Reilly CM, Bruner DW, Mitchell SA et al (2013) A literature synthesis of symptom prevalence and severity in persons receiving active cancer treatment. Support Care Cancer 21:1525–1550. 10.1007/s00520-012-1688-0 23314601 10.1007/s00520-012-1688-0 PMC4299699

[2] Lacouture M, Sibaud V (2018) Toxic side effects of targeted therapies and immunotherapies affecting the skin, oral mucosa, hair, and nails. Am J Clin Dermatol 19:31–39. 10.1007/s40257-018-0384-3 30374901 10.1007/s40257-018-0384-3 PMC6244569

[3] Mooney K, Berry DL, Whisenant M, Sjoberg D (2017) Improving Cancer Care through the patient experience: how to use patient-reported outcomes in clinical practice. Am Soc Clin Oncol Educ Book 37:695–704. 10.14694/edbk_175418 28561689 10.1200/EDBK_175418

[4] Basch E (2014) The rationale for collecting patient-reported symptoms during routine chemotherapy. Am Soc Clin Oncol Educ Book 34:161–165. 10.14694/edbook_am.2014.34.161 10.14694/EdBook_AM.2014.34.161 24857073

[5] Kotronoulas G, Papadopoulou C, Simpson MF et al (2018) Using patient-reported outcome measures to deliver enhanced supportive care to people with lung cancer: feasibility and acceptability of a nurse-led consultation model. Support Care Cancer 26:3729–3737. 10.1007/s00520-018-4234-x 29779057 10.1007/s00520-018-4234-x

[6] Bruera E, Kuehn N, Miller MJ et al (1991) The Edmonton Symptom Assessment System (ESAS): a simple method for the assessment of palliative care patients. J Palliat Care 7:6–9 1714502

[7] Hui D, Bruera E (2016) The Edmonton Symptom Assessment System 25 years later: past, present and future developments. 10.1016/j.jpainsymman.2016.10.370. J Pain Symptom Manage 10.1016/j.jpainsymman.2016.10.370 PMC5337174 28042071

[8] Boddaert M, Douma J, Dijxhoorn F, Bijkerk M (2017) Netherlands quality framework palliative care. In: Utrecht, 2017

[9] van der Baan FH, Koldenhof JJ, de Nijs EJ et al (2020) Validation of the Dutch version of the Edmonton Symptom Assessment System. Cancer Med 9:6111–6121. 10.1002/cam4.3253 32643871 10.1002/cam4.3253 PMC7476846

[10] Wagner LI, Schink J, Bass M et al (2015) Bringing PROMIS to practice: brief and precise symptom screening in ambulatory cancer care. Cancer 121:927–934. 10.1002/cncr.29104 25376427 10.1002/cncr.29104 PMC4352124

[11] IJzerman-Korevaar M, de Graeff A, Heijckmann S et al (2021) Use of a symptom diary on oncology wards. Cancer Nurs 44:E209–E220. 10.1097/NCC.0000000000000792 31990694 10.1097/NCC.0000000000000792

[12] IJzerman-Korevaar M, Snijders TJ, Teunissen SCCM et al (2018) Symptom monitoring in glioma patients: development of the Edmonton Symptom Assessment System glioma module. J Neurosci Nurs 50:381–387. 10.1097/JNN.0000000000000400 30407970 10.1097/JNN.0000000000000400

[13] Koldenhof JJ, van der Baan FH, Verberne EG et al (2022) Patient-reported outcomes during checkpoint inhibition: insight into symptom burden in daily clinical practice. J Pain Symptom Manage 63:997–1005. 10.1016/j.jpainsymman.2022.02.013 35196557 10.1016/j.jpainsymman.2022.02.013

[14] Pinto C, Barone CA, Girolomoni G et al (2011) Management of skin toxicity associated with cetuximab treatment in combination with chemotherapy or radiotherapy. Oncologist 16:228–238. 10.1634/theoncologist.2010-0298 21273511 10.1634/theoncologist.2010-0298 PMC3228080

[15] Fortes BH, Tailor PD, Dalvin LA (2021) Ocular toxicity of targeted anticancer agents. Drugs 81:771–823 33788182 10.1007/s40265-021-01507-z

[16] NVPO (2017) Detecteren behoefte psychosociale zorg. https://richtlijnendatabase.nl/richtlijn/detecteren_behoefte_psychosociale_zorg/meest_geschikte_instrument/welke_instrumenten_zijn_er.html. Accessed 12 Oct 2022

[17] van Trigt I, Boddaert M (2018) Meetinstrumenten in De Palliatieve Zorg

[18] Tuinman MA, Gazendam-Donofrio SM, Hoekstra-Weebers JE (2008) Screening and referral for psychosocial distress in oncologic practice: use of the distress thermometer. Cancer 113:870–878. 10.1002/cncr.23622 18618581 10.1002/cncr.23622

[19] Ownby K, PhD RN, AOCN ACHPN, ANP-BC K (2019) Use of the distress thermometer in clinical practice. J Adv Pract Oncol 10. 10.6004/jadpro.2019.10.2.7 PMC6750919 31538028

[20] Terwee CB, Prinsen CAC, Chiarotto A et al (2018) COSMIN methodology for evaluating the content validity of patient-reported outcome measures: a Delphi study. Qual Life Res 27:1159–1170. 10.1007/s11136-018-1829-0 29550964 10.1007/s11136-018-1829-0 PMC5891557

[21] Mokkink T, Patrick, et al (2010) The COSMIN study reached international consensus on taxonomy, terminology, and definitions of measurement properties for health-related patient-reported. J Clin Epidemiol 63:737–745. 10.1016/j.jclinepi.2010.02.006 20494804 10.1016/j.jclinepi.2010.02.006

[22] Teunissen SCCM, Wesker W, Kruitwagen C et al (2007) Symptom prevalence in patients with incurable cancer: a systematic review. J Pain Symptom Manage 34:94–104. 10.1016/j.jpainsymman.2006.10.015 17509812 10.1016/j.jpainsymman.2006.10.015

[23] Kako J, Kobayashi M, Kanno Y, Ogawa A, Miura T, Matsumoto Y (2018) The optimal cutoff point for expressing revised Edmonton Symptom Assessment System scores as binary data indicating the presence or absence of symptoms. Am J Hospice Palliat Med 35(11):1390–1393. 10.1177/1049909118775660 10.1177/1049909118775660 29734814

[24] Oldenmenger WH, De Raaf PJ, De Klerk C, Van Der Rijt CCD (2013) Cut points on 0–10 numeric rating scales for symptoms included in the Edmonton symptom assessment scale in cancer patients: a systematic review. J Pain Symptom Manage 45:1083–1093. 10.1016/j.jpainsymman.2012.06.007 23017617 10.1016/j.jpainsymman.2012.06.007

[25] Yamaguchi T, Morita T, Nitto A et al (2016) Establishing cutoff points for defining symptom severity using the Edmonton Symptom Assessment System-revised Japanese version. J Pain Symptom Manage 51:292–297. 10.1016/j.jpainsymman.2015.09.011 26598039 10.1016/j.jpainsymman.2015.09.011

[26] Prinsen CAC, Mokkink LB, Bouter LM et al (2018) COSMIN guideline for systematic reviews of patient-reported outcome measures. Qual Life Res 27:1147–1157. 10.1007/s11136-018-1798-3 29435801 10.1007/s11136-018-1798-3 PMC5891568

[27] Robin X, Turck N, Hainard A et al (2011) pROC: an open-source package for R and S + to analyze and compare ROC curves. BMC Bioinformatics 12:77. 10.1186/1471-2105-12-77 21414208 10.1186/1471-2105-12-77 PMC3068975

[28] Mokkink LB, Prinsen CAC, Patrick DL et al (2019) COSMIN study design checklist for patient-reported outcome measurement instruments

[29] Freites-Martinez A, Shapiro J, Goldfarb S et al (2019) CME Part 1: hair disorders in cancer patients. J Am Acad Dermatol 80:1179–1196. 10.1016/j.jaad.2018.03.055.CME 29660422 10.1016/j.jaad.2018.03.055 PMC6186204

[30] Suchonwanit P, McMichael AJ (2018) Alopecia in Association with Malignancy: a review. Am J Clin Dermatol 19:853–865 30088232 10.1007/s40257-018-0378-1

[31] Fabbrocini G, Cameli N, Romano MC et al (2012) Chemotherapy and skin reactions. J Exp Clin Cancer Res 31:1. 10.1186/1756-9966-31-50 10.1186/1756-9966-31-50 PMC3583303 22640460

[32] Petrelli F, Ardito R, Ghidini A et al (2018) Different toxicity of cetuximab and panitumumab in metastatic colorectal cancer treatment: a systematic review and meta-analysis. Oncol (Switzerland) 94:191–199. 10.1159/000486338 10.1159/000486338 29393280

[33] Ostwal V, Kapoor A, Mandavkar S et al (2020) Effect of a structured teaching module including intensive prophylactic measures on reducing the incidence of capecitabine-induced hand-foot syndrome: results of a prospective randomized Phase III Study. Oncologist 25:e1886–e1892. 10.1634/theoncologist.2020-0698 32717127 10.1634/theoncologist.2020-0698 PMC8108045

[34] Kwakman JJM, Elshot YS, Punt CJA, Koopman M (2020) Management of cytotoxic chemotherapy-induced hand-foot syndrome. Oncol Rev 14:57–63 10.4081/oncol.2020.442 PMC7232019 32431787

[35] Borkar DS, Lacouture ME, Basti S (2013) Spectrum of ocular toxicities from epidermal growth factor receptor inhibitors and their intermediate-term follow-up: a five-year review. Support Care Cancer 21:1167–1174. 10.1007/s00520-012-1645-y 23151647 10.1007/s00520-012-1645-y

[36] Yap YS, Kwok LL, Syn N et al (2017) Predictors of hand-foot syndrome and pyridoxine for prevention of capecitabine–induced hand-foot syndrome a randomized clinical trial. JAMA Oncol 3:1538–1545. 10.1001/jamaoncol.2017.1269 28715540 10.1001/jamaoncol.2017.1269 PMC5710192

[37] Ali A, Shah AA, Jeang LJ et al (2022) Emergence of ocular toxicities associated with novel anticancer therapeutics: what the oncologist needs to know. Cancer Treat Rev Apr 105:102376. 10.1016/j.ctrv.2022.102376 10.1016/j.ctrv.2022.102376 35303547

