# Supplementary figures and images for: Validation of 11 added items of the outpatient version of the Utrecht Symptom Diary in patients receiving chemotherapy or targeted therapy
Source: J Patient Rep Outcomes. 2024 Oct 18;8:120. doi: 10.1186/s41687-024-00794-w (PMC11489364; doi:10.1186/s41687-024-00794-w)

**Appendix A: Utrecht Symptom Diary (USD) Outpatient Clinic**


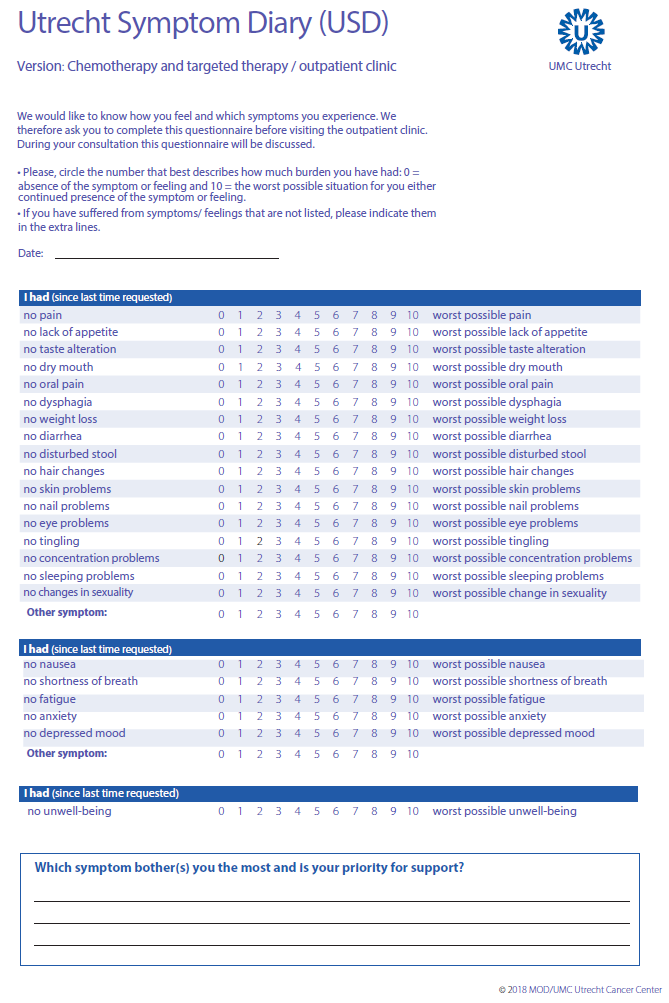

Supplement: Supplementary file 1 — Supplementary Material 1 [file 41687_2024_794_MOESM1_ESM.docx]
