# Supplementary material for: Validation of 11 added items of the outpatient version of the Utrecht Symptom Diary in patients receiving chemotherapy or targeted therapy
Source: J Patient Rep Outcomes. 2024 Oct 18;8:120. doi: 10.1186/s41687-024-00794-w (PMC11489364; doi:10.1186/s41687-024-00794-w)
Supplement: Supplementary file 2 — Supplementary Material 2 [file 41687_2024_794_MOESM2_ESM.docx]

**Treatment regimens**

Adriamycine

adriamycine, cyclofosfamide

Bevacizumab

Bevacizumab, 5FU

Bevacizumab, Carboplatin, Gemcitabine

Bleomycine

Cabazitaxel

capecitabine

cetuximab, carboplatin, capecitabine

cisplatin, 5-FU, cetuximab

Capecitabine, oxaliplatin

Capecitabine, Bevacizumab

Capecitabine, oxaliplatin, bevacizumab

Capacitabine, Oxaliplatin, Trastuzumab

Carboplatin

Carboplatin, etoposide

carboplatin, doxorubicine

carboplatin, paclitaxel (pts daarvóór 4x AC gehad)

Carboplatin, cyclofosfamide, atezolizumab

Carboplatin, Docetaxel

Carboplatin, paclitaxel

Carboplatin, gemcitabine

Carboplatin, Paclitaxel, Bevacizumab

carboplatin, capecitabine

Carboplatin, Vincristine, Cyclofosfamide

Cetuximab

Cisplatin

Cisplatin, Paclitaxel en Bevacizumab

Cisplatin, Gemcitabine

Cisplatin, adriamycine, cyclosfosfamide

Cyclofosfamide, Methotrexaat, Fluorauracil

Dacarbazine

Dactinomycine, Etoposide, Methotrexaat, Folinezuur, Cyclofosfamide

Dactinomycine

Docetaxel, Cyclofosfamide

Docetaxel, Trastuzumab

Docetaxel

Doxorubicine

Doxorubicine en Dacarbazine

Doxorubicine (liposomaal)

Doxorubicine (liposomaal) , bevacizumab

Doxorubicine, Olaratumab

Doxorubicine, Cyclofosfamide

Doxorubicine, Cyclofosfamide, paclitaxel

epirubicine, cisplatin or oxaliplatin, capecitabine

Epirubicine, Cisplatin, Capecitabine + Cisplatin / Capecitabine

epiribucine, oxaliplatin, capecitabine

eribuline

Etoposide, Cisplatin

5-FU, Epirubicine, Cylcofosfamide, Docetaxel

5-FU, Oxaliplatin, Docetaxel

5-FU, bevacizumab

5-FU, irinotecan, panitumumab

5FU, oxaliplatin

5-FU, oxaliplatin, bevacizumab

5-FU, Oxaliplatin, panitumumab

5-FU, oxaliplatin, irinotecan, bevacizumab

5FU, irinotecan liposomaal

gemcitabine

Gemcitabine, Cisplatin

Gemcitabine, capecitabine

Gemcitabine, Docetaxel

Gemcitabine, nab Paclitaxel

Gemcitabine, Oxaliplatin, Paclitaxel

Gemcitabine, Paclitaxel

Irinotecan

Irinotecan, 5-FU

Irinotecan, 5-FU, Bevacizumab

Irinotecan, 5-FU, Oxaliplatin

Methotrexaat

Mitomycine, Capecitabine

olaratumab

Oxaliplatin, Gemcitabine

Oxaliplatin, 5-FU, Bevacizumab

Oxaliplatin, Capecitabine

Oxaliplatin, Capecitabine. Bevacizumab

paclitaxel

Paclitaxel, Carboplatine, Etoposide

Paclitaxel, Bevacizumab

paclitaxel, atezolizumab

Paclitaxel, Carboplatin

panitumumab

pertuzumab

pertuzumab, trastuzumab

Procarbacine, Vincristine, Lomustine

Ramucirumab, Paclitaxel

S-1 (Teysuno®)

Trastuzumab

Trastuzumab, subcutaneously

Trastuzumab_emtansine

Trastuzumab, Docetaxel, Carboplatin

Trastuzumab, Pertuzumab

Trastuzumab, paclitaxel, carboplatin

Trastuzumab, Vinorelbine

Trastuzumab, Capecitabine

Trastuzumab, Paclitaxel

trastuzumab, pertuzumab, carboplatin, Paclitaxel

Vinblastine, methotrexaat

vincristine

vincristine, cyclofosfamide (tbl)

vinorelbine
