# Supplementary material for: Validation of 11 added items of the outpatient version of the Utrecht Symptom Diary in patients receiving chemotherapy or targeted therapy
Source: J Patient Rep Outcomes. 2024 Oct 18;8:120. doi: 10.1186/s41687-024-00794-w (PMC11489364; doi:10.1186/s41687-024-00794-w)
Supplement: Supplementary file 3 — Supplementary Material 3 [file 41687_2024_794_MOESM3_ESM.docx]

Appendix C: Content validity, categorized symptoms

Abdominal cramp

Aggressive

Anal problem

Anger

Anxiety (=fear)

Appetite loss

Ascites

Behavioral change

Bloating

Borborygmi

Breathlessness (=Shortness of breath, dyspnea)

Burping

Chewing problem

Chills

Choking

Cognitive problem (incl. memory problem en concentration problem)

Cold (rhinitis)

Cold feet

Cold hands

Colostoma problem

Constipation

Cough

Cramp (=muscle cramp)

Decubitus

Diarrhoea

Disappointed

Dizziness

Drowsiness

Dry eyes

Dry mouth

Dysphagia

Ear problem

Early satiety

Eczema

Edema

Eye problem (excl. pain, burning eyes)

Fainting

Fatigue (=tiredness, loss of condition, lethargic)

Fecal incontinence

Feeling aroused

Feeling cold

Feeling frustrated

Feeling lonely

Feeling not understood

Feeling rebellious

Fever

Flatulence

Flu

Hair loss

Head tightness

Hearing problem

Heartburn

Hematuria

Hiccup

Hoarseness

Hot flush

Hunger

Hyperacusis

Immobility

Impatient

Instability (incl balance problem, vertigo, imbalance)

Irritability

Itching

Joint problem (not joint pain)

Labile

Lack of emotion

Lethargic

Loss of confidence

Loss of control

Malaise

Mentally exhausted

Motor skill problem

Mouth problem

Muscle weakness

Nail problem

Nasal problem (incl nasal dryness, nasal pain and nasal congestion)

Nausea

Nervousness

Nightmares

Nosebleed

Numbness

Overstimulation

Pain:

- Abdominal pain (incl. stomach pain)
- Anal pain
- Arm pain
- Armpit pain
- Back pain
- Bone pain
- Buttock pain
- Chest pain
- Defecation pain
- Diaphragm pain
- Ear pain
- Esophegeal pain
- Eye pain (incl burning eyes)
- Facial pain
- Flank pain
- Foot pain
- Groin pain
- Hair pain
- Hand pain (incl pain fingers)
- Headache
- Hip pain
- Jaw pain
- Joint pain
- Knee pain
- Leg pain
- Lymph gland pain
- Menstrual pain
- Mouth pain
- Muscle pain
- Neck pain
- Nerve pain
- Salivary gland pain
- Scalp pain
- Scar pain
- Scrotum pain
- Shoulder pain
- Sinus problem
- Sore throat
- Tooth pain (incl. tooth ache)
- Vulvar pain

Nosebleed

Overstimulation

Powerless

Restless legs

Restlessness

Palpitation

Petechiae

Phlegm

Rectal bleeding

Restlessness

Regurgitation

Retching

Sadness

Salivation

Seizure

Sexual problem

Skin acne

Skin blisters

Skin discoloration

Skin dryness

Skin Hand-foot syndrome

Skin irritation

Skin rash

Skin redness

Skin sensitivity

Skin shedding

Sleeping problem

Smell problem

Speech problem

Stiffness

Stress

Sweating

Swallowing problem

Swollen glands

Swollen neck

Swollen tongue

Taste problem

Tense

Thirst

Tingling (=paresthesia)

Tinnitus

Tremor

Trismus

Twitching

Uncertainty

Urination problem

Vaginal problem (incl bleeding, vaginal discharge)

Vertigo

Vision problem

Vomiting

Weight gain

Weight loss

Worried

Wound
